# Effectiveness of Ascophyllum nodosum and Fucus vesiculosus on Metabolic Syndrome Components: A Real-World, Observational Study

**DOI:** 10.1155/2021/3389316

**Published:** 2021-09-30

**Authors:** Antonio Nicolucci, Maria Chiara Rossi, Massimiliano Petrelli

**Affiliations:** ^1^Center for Outcomes Research and Clinical Epidemiology (CORESEARCH), Pescara, Italy; ^2^Clinic of Endocrinology and Metabolic Diseases, Azienda Ospedaliero-Universitaria “Ospedali Riuniti di Ancona”, Ancona, Italy

## Abstract

**Introduction:**

Gdue is a nutraceutical obtained from the association of two marine algae, Ascophyllum nodosum and Fucus vesiculosus, in addition to chromium picolinate, which could be useful for the treatment of dysglycemia, overweight, and the other components of the metabolic syndrome. The aim of the study was to assess the real-world effectiveness and safety of Gdue when administered to subjects with one or more components of the metabolic syndrome.

**Methods:**

A longitudinal, retrospective, observational study, conducted among primary care physicians, nutritionists, and specialists from various disciplines. The impact of 180 days of administration of Gdue was assessed on body weight, waist circumference, fasting blood glucose, HbA1c, lipid profile, and blood pressure levels. The likelihood of experiencing a first major cardiovascular event over ten years was estimated using Italian risk charts. General linear models for repeated measures were applied to assess changes in the parameters of interest during the follow-up. Results are expressed as estimated marginal means with their 95% confidence interval.

**Results:**

Overall, 505 patients were enrolled by 282 physicians. After 6 months of treatment with Gdue, body weight was reduced on average by 7.3 kg (-8.0; -6.6), waist circumference by 7.5 cm (-8.2; -6.8), fasting blood glucose by 16.3 mg/dL (-17.8; -14.7), HbA1c by 0.55% (-0.62; -0.49), systolic and diastolic blood pressure by 7.1 mmHg (-8.3; -6.0) and 4.2 mmHg (-5.0; -3.5), respectively, LDL cholesterol by 18.2 mg/dL (-21.2; -15.3), and triglycerides by 39 mg/dL (-45; -32). HDL cholesterol was significantly increased by 2.9 mg/dL (0.7; 5.0). The 10-year risk of cardiovascular events significantly decreased by 1.8%, corresponding to a relative risk reduction of 27.7%.

**Conclusion:**

Our real-world study shows that 6 months of treatment with Gdue have an impact on all the components of the metabolic syndrome, thus offering the potential for decreasing the cardiovascular risk associated with metabolic syndrome.

## 1. Introduction

Metabolic syndrome has become a major health problem in most of the modern world and is characterized by abdominal obesity, insulin resistance, dysglycemia, hypertension, and hyperlipidemia [1, 2]. The major determinant of this condition is the increasing adoption of unhealthy lifestyles, including the consumption of high calorie-low fiber foods and the decrease in both work and recreational physical activity. The metabolic syndrome feeds into the spread of type 2 diabetes, coronary diseases, stroke, and other disabilities.

Two marine algae, Ascophyllum nodosum and Fucus vesiculosus, could be useful for the treatment of dysglycemia, overweight, and the other components of the metabolic syndrome [3–5]. Ascophyllum nodosum is a brown alga of the Fucaceae family that proliferates on the coasts of the North Atlantic Ocean. Fucus vesiculosus belongs to the Fucaceae family and is present in all cold waters [6]. Gdue is an algal extract obtained from the association of Ascophyllum nodosum and Fucus vesiculosus, in addition to chromium picolinate. The two algae, present in Gdue according to a ratio of 95 : 5, are able to improve the balance of body weight and stimulate general metabolism, in particular that of lipids and carbohydrates [4, 5, 7–12]. The phlorotannins contained in algae exert a noncompetitive and reversible blocking action of the *α*-amylase and *α*-glucosidase enzymes in the intestine, causing a slowdown in the absorption and digestion of carbohydrates and demonstrating an important antihyperglycemic action in vivo, in particular on postprandial hyperglycemia [13–17]. In addition, brown seaweeds are characterized by the presence of different proteins, lipids, carbohydrates, vitamins, and minerals. In recent years, polyphenols, sulfated polysaccharides, carotenoids, and polyunsaturated fatty acids (PUFAs) have been evaluated as adjuvants for the treatment and prevention of metabolic syndrome-related diseases [18]. Furthermore, brown seaweeds contain many useful minerals and indigestible polysaccharides, able to affect the digestion and absorption of starch and other complex carbohydrates [18].

Chromium is an essential trace element involved in the metabolism of carbohydrates, lipids, and proteins and is a necessary cofactor for many insulin functions, promoting the binding to its receptor in muscle cells, adipocytes, and hepatocytes and also promoting the phosphorylation of receptors [19–21].

In streptozotocin-induced diabetic animals, administration of chromium picolinate decreased plasma glucose levels, normalized glycogen content in the liver, increased the activity of glycolytic enzymes (glucokinase, phosphofructokinase, and pyruvate kinase), and suppressed the activity of gluconeogenic enzymes (glucose-6-phosphatase and phosphoenolpyruvate carboxykinase) in the liver [22]. The inhibition of resistin secretion via activation of AMPK in normal and insulin-resistant 3T3-L1 adipocytes has also been suggested as an additional mechanism of action [23].

At the oral dose of 250-500 mg, Gdue reaches levels 25-50 times higher in intestinal fluids than in vitro inhibitory concentrations, completely, uncompetitively, and reversibly inhibiting carbohydrate-degrading enzymes. Preclinical studies have confirmed this mechanism of action [7, 8], responsible for a marked reduction in the variability in metabolic response, with the lowering of the peaks of postprandial glycemia, less oxidative stress, and lower levels of insulinemia, with consequent lower risk of pancreatic *β*-cell exhaustion.

Animal studies also suggest a positive effect of Ascophyllum nodosum and Fucus vesiculosus on liver steatosis, frequently associated with the metabolic syndrome [10].

Open-label or double-blind controlled trials have shown how Gdue reduces the glycemic index of ingested foods, postprandial glycemic peaks, and the consequent insulin response [4, 9–11]. With Gdue, a reduction in HbA1c, fasting plasma glucose (FPG), postprandial plasma glucose (PPG), and HOMA-IR was observed compared to placebo. A reduction in C-Reactive Protein (CRP) and TNF-*α* levels, evaluated as endothelial damage markers [11], was also evidenced. As for lipid parameters, a significant reduction vs. baseline in total cholesterol, LDL cholesterol, and triglycerides levels was documented [4]. Furthermore, a reduction in abdominal circumference has been consistently observed in the studies mentioned above [5, 11].

Assessing whether the results of these clinical trials are applicable to the broader range of patient populations treated under routine clinical practice conditions and in different settings is of importance to determine the magnitude of effectiveness of Gdue use.

The aim of the study was to assess the real-world effectiveness and safety of Gdue when administered to subjects with one or more components of the metabolic syndrome. The impact of 180 days of administration of Gdue was assessed on fasting blood glucose, HbA1c, body weight, waist circumference, lipid profile, and blood pressure levels.

## 2. Materials and Methods

### 2.1. Design

This is a longitudinal, retrospective, observational study, conducted among primary care physicians, nutritionists, and specialists from various disciplines.

Patients were included if they fulfilled the following eligibility criteria:

1. Male or female patients aged ≥18 years
2. Presence of one or more components of the metabolic syndrome, according to the IDF definition [2], including waist circumference ≥ 94 cm in males and ≥80 cm in females, triglycerides > 150 mg/dL, HDL cholesterol < 40 mg/dL in males and <50 mg/dL in females, systolic blood pressure ≥ 130 mmHg or diastolic blood pressure ≥ 85 mmHg or antihypertensive therapy, impaired fasting glucose (IFG; 110-125 mg/dL), or type 2 diabetes (T2DM)
3. Patients treated with Gdue. The decision to prescribe Gdue was based on clinical judgment and was independent of study participation

Gdue was generally administered at the recommended dose of 2-3 tablets per day at meals as integration of a dietary prescription.

At baseline (start of Gdue) and after 90 days and 180 days of treatment, the following information was collected from clinical records on an ad hoc form: age, gender, body weight, waist circumference, fasting blood glucose, HbA1c, blood pressure, total cholesterol, HDL cholesterol, LDL cholesterol, and triglycerides. Information on comorbidities and concomitant treatments was also collected. Participating physicians were requested to report any adverse event registered during the follow-up.

In a subgroup of 89 patients, information on fasting insulin levels was also available at baseline and during the follow-up. In these patients, HOMA-IR was also calculated as an index of insulin resistance. Data collected were those available in medical records and prescribed according to routine clinical practice, and no laboratory test was performed specifically for the study.

### 2.2. Statistical Analysis

Descriptive data were summarized as mean and standard deviation (continuous variables) or counts and percentages (categorical variables).

Patient characteristics at baseline were compared by gender using the Mann-Whitney *U*-test for continuous variables and the chi-square test for categorical variables.

General linear models for repeated measures were applied to assess changes in the parameters of interest during the follow-up. Results are expressed as estimated marginal means with their 95% confidence interval (95% CI). Mean changes of each parameter were also estimated by tertile of the parameter at baseline. All tests are two-sided, and a *P* value < 0.05 was considered for statistical significance.

The likelihood of experiencing a first major cardiovascular event (myocardial infarction or stroke) over the following ten years was estimated using the Progetto Cuore Italian risk charts [24].

Statistical analyses were performed using the SPSS software ver. 23.0 (IBM, Armonk, NY, USA).

## 3. Results

Overall, 505 patients were enrolled by 282 physicians (range 1-8). Among participating physicians, 65.5% were general practitioners, 10.9% were nutritionists, 8.1% were endocrinologists, and 15.5% were from other specialties (cardiology, nephrology, gastroenterology, and internal medicine). The characteristics of the patients enrolled by gender are reported in Table 1. For 14 patients, the gender was not reported. Male patients showed significantly higher values than females in terms of age, body weight, waist circumference, fasting blood glucose, and systolic blood pressure and lower HDL cholesterol levels. The prevalence of T2DM and hypertension, as well as the 10-year cardiovascular risk, was also significantly higher in men than in women.

The proportion of patients with availability of information at baseline varied between 98.6% for body weight and 49.9% for total cholesterol; the availability of information relative to 180 days ranged from 88.9% for body weight to 41.8% for total cholesterol (supplementary table 1). Overall, 138 patients had complete data for all the variables investigated both at baseline and after 180 days, allowing the estimation of the 10-year cardiovascular risk. The characteristics of patients with full data and those with incomplete data are reported in supplementary table 2. Patients with complete data were significantly older than those with incomplete data (58.5 ± 13.7 vs. 56.0 ± 12.4 years; *P* = 0.03) and more likely to have hypertension (51.4% vs. 38.1%; *P* = 0.007). No other statistically significant differences emerged for all the other variables investigated.

**Figure 1:**
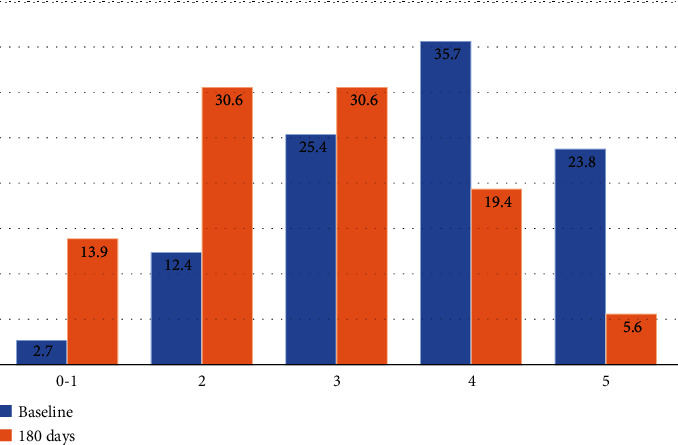
Number of components of the metabolic syndrome at baseline and after 180 days of treatment with Gdue. Components of the metabolic syndrome according to the IDF criteria [2]: waist circumference (M ≥ 94 cm; F ≥ 80 cm); triglycerides > 150 mg/dL; HDL cholesterol (M < 40 mg/dL; F < 50 mg/dL); blood pressure (systolic blood pressure ≥ 130 mmHg or diastolic blood pressure ≥ 85 mmHg or antihypertensive treatment); impaired fasting glucose or type 2 diabetes.

Table 2 reports the longitudinal changes in the parameters of interest. Both after 90 days and 180 days, a significant improvement in all the parameters was documented. In particular, after 6 months of treatment with Gdue, body weight was reduced on average by 7.3 kg, waist circumference by 7.5 cm, fasting blood glucose by 16.3 mg/dL, HbA1c by 0.55%, systolic and diastolic blood pressure by 7.1 mmHg and 4.2 mmHg, respectively, LDL cholesterol by 18.2 mg/dL, and triglycerides by 39 mg/dL. HDL cholesterol was significantly increased by 2.9 mg/dL. A significant reduction in fasting insulin levels and HOMA-IR was also documented. These changes translated into a significant reduction in the number of components of the metabolic syndrome (Figure 1). The 10-year risk of cardiovascular events significantly decreased by 1.8%, corresponding to a relative risk reduction of 27.7%.

The analysis was also performed by gender (supplementary table 3). All the parameters considered significantly improved in men and women, with the only exception of HDL cholesterol levels, which increased significantly in men only (+5.2 mg/dL after 180 days; *P* < 0.0001).

The analyses by tertiles of the baseline values of each parameter further documented that the benefits were significant at any baseline level of body weight, waist circumference, fasting blood glucose, HbA1c, and triglycerides, while for blood pressure and LDL cholesterol, no clear benefit was documented in patients in the lower tertile; similarly, no significant improvement in HDL cholesterol levels was documented in patients in the upper tertile (Table 3). The more the values of the different parameters departed from normality, the larger was the reduction documented.

Study results were not affected when data were analyzed according to the dietary prescription made to the patients (free diet, balanced diet with -500 Kcal compared to the estimated Total Daily Energy Expenditure; supplementary table 4).

Mild gastrointestinal side effects (aerophagia, heartburn, diarrhea, nausea, and abdominal pain) were reported by 3.4% (*N* = 17) of the patients; moderate gastrointestinal side effects (diarrhea and significant gastrointestinal tension) were referred by 0.6% (*N* = 3) of the patients.

## 4. Discussion

### 4.1. Main Findings

Real-world studies represent an important complement to randomized clinical trials, providing information on the effectiveness of a treatment when administered to unselected populations and in different settings.

Our study shows that, under routine clinical practice conditions, the treatment with Gdue for 6 months produced substantial benefits on all the components of the metabolic syndrome, including fasting plasma glucose, HbA1c, body weight, waist circumference, blood pressure, and lipid profile. A reduction in fasting plasma insulin levels and HOMA-IR was also documented. The benefits were already evident after three months of treatment and further increased at six months.

Of note, significant benefits were obtained even in patients in the lower tertile of each parameter, although the magnitude of the benefit increased with increasing values at baseline. These data suggest that Gdue can play an important role not only in patients with overt diabetes, hypertension, or dyslipidemia but also in earlier stages of the metabolic syndrome.

The benefits on the components of the metabolic syndrome translated into a significant decrease in the estimated 10-year risk of major cardiovascular events, with a relative risk reduction of 27.7%.

### 4.2. Comparison with Existing Evidence

In two double-blind, placebo-controlled trials, the treatment with Gdue for 6 months produced a significant decrease in FBG and HbA1c levels in individuals with dysglycemia or T2DM [5, 11]. In line with these studies, we documented a relevant reduction in FBG (-16.3 mg/dL) and HbA1c levels (-0.55%). Of note, the average reduction in FBG levels was -23.5 mg/dL among patients with FBG values > 125 mg/dL at baseline, and the reduction in HbA1c levels reached -0.86% in individuals with HbA1c > 7.0% at baseline.

The beneficial effects of Gdue on waist circumference were also previously documented in a single-arm, 6-month study involving 50 overweight or obese patients [3], which showed an average reduction in waist circumference from 112 ± 17 cm at baseline to 105 ± 13 cm after 6 months of treatment. Similarly, in a single-arm study involving 47 patients with DM2 waist circumference was reduced from 102 ± 4 cm at baseline to 100 ± 5 cm after six months of treatment (*P* < 0.05) [4]. In our study, the average reduction in waist circumference was 7.5 cm after six months, without major differences by gender. Of note, no significant reduction in either body weight or waist circumference was documented in the two randomized trials previously cited [5, 11].

The impact of Gdue therapy on lipid profile was assessed in a randomized, double-blind placebo-controlled trial involving 175 individuals with T2DM [11]. In this trial, the treatment with Gdue was not associated with significant reductions vs. placebo in the levels of total cholesterol, LDL cholesterol, HDL cholesterol, and triglycerides. On the other hand, the single-arm study involving 47 patients with DM2 previously cited [10] documented a significant reduction in total cholesterol (from 198 ± 8 mg/dL to 180 ± 5 mg/dL; *P* < 0.001), LDL cholesterol (from 105 ± 4 mg/dL to 95 ± 2 mg/dL; *P* < 0.05), and triglyceride levels (from 167 ± 6 mg/dL to 148 ± 8 mg/dL; *P* < 0.05) and a significant increase in HDL cholesterol levels (from 50 ± 4 mg/dL to 55 ± 4 mg/dL; *P* < 0.05). In our study, we found a statistically significant effect on all the parameters of the lipid profile, with an average reduction of 18.2 mg/dL for LDL cholesterol and 39.0 mg/dL for triglycerides, while HDL cholesterol was significantly increased by 5.2 mg/dL in men.

To our knowledge, no previous studies have assessed the impact of Gdue therapy on blood pressure. We documented a significant reduction in systolic and diastolic blood pressure, evident after three months and even larger after six months, with an average reduction of 7.1 mmHg for systolic blood pressure and 4.2 mmHg for diastolic blood pressure. The effect on blood pressure can represent the consequence of weight loss; in fact, it is well established that weight loss is associated with a parallel decrease in systolic and diastolic blood pressure [25].

Finally, a reduction vs. baseline in fasting insulin levels and HOMA index after 6 months of treatment with Gdue was described in the study by De Martin et al. [3], while a significant reduction vs. placebo in HOMA index, but not fasting insulin, was documented in a randomized trial conducted in individuals with dysglycemia [11].

The study has strengths and limitations. Among the strengths, the large number of patients and healthcare professionals involved provides a realistic picture of the effectiveness of Gdue under routine clinical practice conditions. Also, the study allowed to assess the impact of the treatment on a large array of different parameters of the metabolic syndrome.

Among the limitations, it should be emphasized that follow-up data were not available for all patients. Also, BMI could not be calculated given the lack of information on height in most of the patients. However, it should also be noted that the degree of missingness was modest for some key variables such as FBG, body weight, and waist circumference. Furthermore, baseline characteristics of individuals with full data and those with missing data were largely superimposable, with the only exception of an older age and a higher prevalence of hypertension among those with all the information available. Another limitation is represented by the lack of a control group; in addition to the therapy with Gdue, additional aspects of care may have influenced the study results. In this respect, it is worth mentioning that study results were not affected when data were analyzed according to the dietary prescription made to the patients (free diet and balanced diet).

## 5. Conclusions

In conclusion, our real-world study shows that 6 months of treatment with Gdue have an impact on all the components of the metabolic syndrome, thus offering the potential for decreasing the cardiovascular risk associated with metabolic syndrome. A significant reduction in the levels of key risk factors was documented even in patients in the lower tertile of the distribution of each variable, suggesting that also individuals with initial, mild elevation of FBG, blood pressure, or lipid parameters can benefit from this therapeutic approach.

## Figures and Tables

**Table 1:** Patient characteristics by gender (data are mean ± SD or %).

Characteristic	Males	Females	*P*
*N*	214	277	
Age (years)	58.2 ± 12.6	55.7 ± 12.9	0.006
Body weight (kg)	97.6 ± 16.4	85.9 ± 17.0	<0.0001
Waist circumference (cm)	115.2 ± 16.1	103.3 ± 17.5	<0.0001
Fasting blood glucose (mg/dL)	126.9 ± 24.4	118.1 ± 23.3	<0.0001
HbA1c (%)			
Patients with T2DM	7.4 ± 1.0	7.2 ± 0.8	0.28
Patients without T2DM	6.4 ± 0.8	6.3 ± 0.8	0.22
Systolic blood pressure (mmHg)	137.2 ± 14.4	132.5 ± 14.2	0.0001
Diastolic blood pressure (mmHg)	83.4 ± 9.4	81.9 ± 11.1	0.06
Total cholesterol (mg/dL)	215.3 ± 40.8	221.3 ± 41.0	0.31
HDL cholesterol (mg/dL)	45.5 ± 12.0	53.9 ± 20.9	<0.0001
LDL cholesterol (mg/dL)	130.4 ± 36.9	135.3 ± 37.4	0.36
Triglycerides (mg/dL)	187.4 ± 89.7	166.8 ± 66.2	0.07
Fasting insulin (*μ*u/mL)	25.8 ± 16.3	27.8 ± 33.4	0.37
HOMA-IR	7.6 ± 4.7	10.4 ± 8.0	0.18
Diabetes (%)	32.7	22.7	0.01
Hypertension (%)	47.7	37.9	0.03
Dyslipidemia (%)	16.8	16.6	0.95
Cardiovascular disease (%)	4.7	5.4	0.71
Number of components of the metabolic syndrome			0.49
1	2.1	2.4	
2	7.2	15.0	
3	27.8	27.6	
4	39.2	33.9	
5	23.7	21.3	
10-year CVD risk (%)	16.7 ± 16.3	5.2 ± 8.8	<0.0001

**Table 2:** Estimated marginal means (95% CI) of the parameters of interest at baseline and their change after 90 days and 180 days of treatment.

Parameter	Baseline value	Change at 90 days	*P* value (90 days vs. baseline)	Change at 180 days	*P* value (180 days vs. baseline)
Weight (kg)	91.3 [89.5; 93.0]	-4.6 [-5.0; -4.2]	<0.0001	-7.3 [-8.0; -6.6]	<0.0001
Waist circumference (cm)	107.9 [105.9; 109.9]	-4.3 [-4.8; -3.8]	<0.0001	-7.5 [-8.2; -6.8]	<0.0001
Fasting blood glucose (mg/dL)	122.6 [120.1; 125.0]	-10.6 [-11.8; -9.3]	<0.0001	-16.3 [-17.8; -14.7]	<0.0001
HbA1c (%)	6.66 [6.55; 6.77]	-0.33 [-0.39; -0.27]	<0.0001	-0.55 [-0.62; -0.49]	<0.0001
Systolic blood pressure (mmHg)	134 [133; 136]	-4.8 [-3.7; -5.8]	<0.0001	-7.1 [-8.3; -6.0]	<0.0001
Diastolic blood pressure (mmHg)	82 [81; 83]	-2.6 [-3.2; -2.0]	<0.0001	-4.2 [-5.0; -3.5]	<0.0001
LDL cholesterol (mg/dL)	131 [127; 136]	-11.1 [-13.4; -9.0]	<0.0001	-18.2 [-21.2; -15.3]	<0.0001
HDL cholesterol (mg/dL)	49.7 [47.0; 52.3]	1.5 [-0.5; 3.5]	0.15	2.9 [0.7; 5.0]	<0.0001
Triglycerides (mg/dL)	185 [174; 196]	-23 [-34; -12]	<0.0001	-39 [-45; -32]	0.009
Fasting plasma insulin (*μ*u/mL)	28.0 [21.8; 34.2]	-3.9 [-6.4; -1.4]	0.002	-5.7 [-8.4; -3.0]	<0.0001
HOMA-IR	8.2 [6.2; 10.2]	-1.6 [-2.3; -0.9]	<0.0001	-2.5 [-3.3; -1.7]	<0.0001
10-year cardiovascular risk (%)	6.5 [5.0; 7.9]	-1.2 [-1.5; -0.8]	<0.0001	-1.8 [-2.4; -1.2]	<0.0001

**Table 3:** Estimated marginal means (95% CI) of the parameters of interest at baseline and their change after 90 days and 180 days of treatment, by tertiles of baseline values or clinically meaningful classes (for FBG and HbA1c).

Parameter	Baseline value	Change at 90 days	*P* value (90 days vs. baseline)	Change at 180 days	*P* value (180 days vs. baseline)
Weight (kg)					
I	75.2 [73.6; 76.8]	-3.1 [-3.5; -2.7]	<0.0001	-4.7 [-5.5; -3.9]	<0.0001
II	87.9 [86.8; 89.1]	-3.7 [-4.1; -3.2]	<0.0001	-6.2 [-6.8; -5.5]	<0.0001
III	109.3 [106.6; 112.0]	-6.9 [-8.5; -5.3]	<0.0001	-10.8 [-12.7; -9.0]	<0.0001
Waist circumference (cm)					
I	91.8 [89.8; 93.8]	-3.1 [-3.5; -2.6]	<0.0001	-5.4 [-6.1; -4.8]	<0.0001
II	106.2 [105.0; 107.5]	-4.5 [-5.1; -3.9]	<0.0001	-8.1 [-9.0; -7.3]	<0.0001
III	126.9 [124.8; 129.1]	-5.7 [-6.9; -4.5]	<0.0001	-9.5 [-11.1; -7.8]	<0.0001
Fasting blood glucose (mg/dL)					
<110	89.5 [85.2; 93.9]	-2.1 [-3.7; -0.6]	0.007	-3.1 [-5.5; -0.7]	0.013
110-125	112.8 [11.7; 114.0]	-8.1 [-9.2; -7.1]	<0.0001	-12.8 [-14.1; -11.5]	<0.0001
>125	141.8 [138.5; 145.1]	-15.5 [-17.9; -13.0]	<0.0001	-23.5 [-26.3; -20.7]	<0.0001
HbA1c (%)					
<6.0	5.48 [5.36-5.59]	-0.18 [-0.26; -0.10]	<0.0001	-0.27 [-0.38; -0.16]	<0.0001
6.0-6.5	6.25 [6.21-6.29]	-0.20 [-0.25; -0.15]	<0.0001	-0.41 [-0.49; -0.32]	<0.0001
6.6-7.0	6.83 [6.80-6.86]	-0.38 [-0.49; -0.28]	<0.0001	-0.58 [-0.69; -0.47]	<0.0001
>7.0	7.74 [7.60-7.87]	-0.52 [-0.67; -0.37]	<0.0001	-0.86 [-1.00; -0.72]	<0.0001
Systolic blood pressure (mmHg)					
I	117.2 [115.2; 119.3]	0.3 [-1.4; 2.1]	0.69	0.17 [-2.8; 1.2]	0.86
II	135.1 [134.3; 135.9]	-4.7 [-5.8; -3.7]	<0.0001	-6.6 [-7.8; -5.5]	<0.0001
III	152.4 [150.0; 154.8]	-10.3 [-12.3; -8.4]	<0.0001	-16.3 [-18.7; -12.4]	<0.0001
Diastolic blood pressure (mmHg)					
I	70.1 [68.5; 71.6]	0.9 [-0.5; 2.2]	0.22	0.9 [-0.8; 2.6]	0.29
II	81.5 [81.2; 81.9]	-2.2 [-2.9; -1.5]	<0.0001	-3.7 [-4.5; -2.8]	<0.0001
III	93.1 [90.2; 95.9]	-6.0 [-7.1; -4.9]	<0.0001	-9.4 [-10.6; -8.1]	<0.0001
LDL cholesterol (mg/dL)					
I	95.5 [91.2; 97.7]	-2.3 [-6.2; 1.7]	0.25	-3.7 [-8.3; 0.9]	0.11
II	131.4 [129.5; 133.3]	-11.3 [-13.9; -8.7]	<0.0001	-18.6 [-21.8; -15.3]	<0.0001
III	168.9 [163.2; 174.6]	-19.9 [-23.9; -16.0]	<0.0001	-32.5 [-37.7; -27.2]	<0.0001
HDL cholesterol (mg/dL)					
I	37.0 [35.9; 38.2]	4.0 [2.8; 5.3]	<0.0001	6.6 [5.1; 8.1]	<0.0001
II	47.1 [46.1; 48.1]	3.0 [1.7; 4.4]	<0.0001	4.8 [3.2; 6.4]	<0.0001
III	67.6 [61.5; 73.8]	-3.0 [-9.2; 3.1]	0.33	-3.6 [-9.8; 2.6]	0.25
Triglycerides (mg/dL)					
I	110.0 [104.4; 115.6]	-4.6 [-8.6; -0.6]	0.025	-8.1 [-12.6; -3.7]	0.001
II	168.5 [165.1; 171.9]	-20.1 [25.3; -14.9]	<0.0001	-25.8 [-32.2; -19.4]	<0.0001
III	254.4 [235.0; 273.7]	-39.0 [-67.0; -10.9]	<0.0001	-72.9 [-86.0; -59.9]	<0.0001

## Data Availability

All data generated or analyzed during this study are included in this published article.

## Supplementary Materials

Supplementary Materials Supplementary Table 1: availability of information at baseline and at 6 months follow-up. Supplementary Table 2: patient characteristics by availability of all the information at baseline and after 180 days of observation (data are mean ± SD or %). Supplementary Table 3: estimated marginal means (95% CI) of the parameters of interest at baseline and their change after 90 days and 180 days of treatment by gender. Supplementary Table 4: estimated marginal means (95% CI) of the parameters of interest at baseline and after 90 days and 180 days of treatment, according to the type of diet (free diet, *N* = 267; balanced diet with -500 Kcal compared to the estimated Total Daily Energy Expenditure, *N* = 238).
