# Supplementary material for: Effectiveness of Ascophyllum nodosum and Fucus vesiculosus on Metabolic Syndrome Components: A Real-World, Observational Study
Source: J Diabetes Res. 2021 Sep 30;2021:3389316. doi: 10.1155/2021/3389316 (PMC8497120; doi:10.1155/2021/3389316)
Supplement: Supplementary Materials — Supplementary Table 1: availability of information at baseline and at 6 months follow-up. Supplementary Table 2: patient characteristics by availability of all the information at baseline and after 180 days of observation (data are mean ± SD or %). Supplementary Table 3: estimated marginal means (95% CI) of the parameters of interest at baseline and their change after 90 days and 180 days of treatment by gender. Supplementary Table 4: estimated marginal means (95% CI) of the parameters of interest at baseline and after 90 days and 180 days of treatment, according to the type of diet (free diet, N = 267; balanced diet with -500 Kcal compared to the estimated Total Daily Energy Expenditure, N = 238). [file 3389316.f1.docx]

Supplementary table 1. Availability of information at baseline and at 6 months follow-up.

| Characteristic | Data availability N (%) | |
| --- | --- | --- |
|  | Baseline | 6 months |
| Body weight | 498 (98.6) | 449 (88.9) |
| Waist circumference | 447 (88.5) | 402 (79.6) |
| Fasting blood glucose | 475 (94.1) | 412 (81.6) |
| HbA1c | 360 (71.3) | 318 (63.0) |
| Systolic blood pressure | 338 (66.9) | 294 (58.2) |
| Diastolic blood pressure | 338 (66.9) | 294 (58.2) |
| Total cholesterol | 252 (49.9) | 211 (41.8) |
| HDL cholesterol | 304 (60.2) | 248 (49.1) |
| LDL cholesterol | 291 (57.6) | 246 (48.7) |
| Triglycerides | 311 (61.6) | 254 (50.3) |

Supplementary table 2. Patient characteristics by availability of all the information at baseline and after 180 days of observation (data are mean±SD or %)

| Characteristic | Data availability | | p |
| --- | --- | --- | --- |
|  | Yes | No |  |
| N | 138 | 360 |  |
| Age (years) | 58.5±13.7 | 56.0±12.4 | 0.03 |
| Gender (% males) | 44.5 | 43.2 | 0.79 |
| Body weight (kg) | 92.5±16.9 | 90.5±18.1 | 0.18 |
| Waist circumference (cm) | 107.5±15.7 | 109.2±18.6 | 0.36 |
| Fasting blood glucose (mg/dl) | 124.6±27.2 | 120.9±22.8 | 0.20 |
| HbA1c (%) | 6.7±1.0 | 6.6±0.9 | 0.15 |
| Systolic blood pressure (mmHg) | 136.5±14.2 | 133.4±14.4 | 0.15 |
| Diastolic blood pressure (mmHg) | 81.7±8.1 | 83.3±11.6 | 0.31 |
| Total cholesterol (mg/dl) | 220.3±38.8 | 217.0±42.3 | 0.66 |
| HDL cholesterol (mg/dl) | 49.5±14.3 | 51.0±20.3 | 0.74 |
| LDL cholesterol (mg/dl) | 134.0±32.2 | 132.5±40.2 | 0.51 |
| Triglycerides (mg/dl) | 186.4±87.9 | 166.3±67.2 | 0.06 |
| Diabetes (%) | 31.9 | 25.1 | 0.12 |
| Hypertension (%) | 51.4 | 38.1 | 0.007 |
| Dyslipidemia (%) | 15.9 | 16.9 | 0.80 |
| Cardiovascular disease (%) | 5.8 | 4.9 | 0.69 |
| Number of components of the metabolic syndrome  1  2  3  4  5 | 2.4  11.8  22.8  36.2  26.8 | 2.1  11.3  34.0  36.1  16.5 | 0.28 |

Supplementary table 3. Estimated marginal means [95% CI] of the parameters of interest at baseline and their change after 90 days and 180 days of treatment by gender

Females

| Parameter | Baseline  value | Change  at 90 days | P value  (90 days vs.  baseline) | Change  at 180 days | P value  (180 days vs.  baseline) |
| --- | --- | --- | --- | --- | --- |
| Weight (Kg) | 85.8 [83.5; 88.1] | -4.8 [-5.8; -3.8] | <0.0001 | -7.3 [-8.5; -6.2] | <0.0001 |
| Waist circumference (cm) | 103.1 [100.6; 105.5] | -4.2 [-4.9; -3.5] | <0.0001 | -7.4 [-8.4; -6.5] | <0.0001 |
| Fasting blood glucose (mg/dl) | 118.8 [115.5; 122.1] | -10.0 [-11.6; -8.3] | <0.0001 | -15.2 [-17.2; -13.2] | <0.0001 |
| HbA1c (%) | 6.56 [6.42; 6.70] | -0.32 [-0.37; -0.27] | <0.0001 | -0.54 [-0.63; -0.46] | <0.0001 |
| Systolic blood pressure (mmHg) | 133 [131; 135] | -4.6 [-6.1; -3.2] | <0.0001 | -6.9 [-8.6; -5.2] | <0.0001 |
| Diastolic blood pressure (mmHg) | 82 [80; 83] | -2.7 [-3.6; -1.9] | <0.0001 | -4.6 [-5.6; -3.6] | <0.0001 |
| LDL cholesterol (mg/dl) | 132 [125; 138] | -9.8 [-12.7; -7.0] | <0.0001 | -17.2 [-21.1; -13.3] | <0.0001 |
| HDL cholesterol (mg/dl) | 54.2 [50.1; 58.3] | 0.4 [-3.1; 3.8] | 0.83 | 1.3 [-2.3; 4.8] | 0.48 |
| Triglycerides (mg/dl) | 175 [163; 186] | -18 [-35; -2] | 0.036 | -38 [-45; -30] | <0.0001 |
| Fasting plasma Insulin (μu/ml) | 29.7 [20.1; 39.4] | -4.1 [-7.9; -0.4] | 0.032 | -5.8 [-9.8; -1.7] | 0.006 |
| HOMA-IR | 8.7 [5.6; 11.8] | -1.7 [-2.7; -0.7] | 0.001 | -2.6 [-3.7; -1.5] | <0.0001 |
| 10-year cardiovascular risk (%) | 5.7 [3.5; 7.9] | -0.8 [-1.3; -0.4] | 0.001 | -1.3 [-1.9; -0.7] | <0.0001 |

Males

| Parameter | Baseline  value | Change  at 90 days | P value  (90 days vs.  baseline) | Change  at 180 days | P value  (180 days vs.  baseline) |
| --- | --- | --- | --- | --- | --- |
| Weight (Kg) | 98.3 [95.8; 100.7] | -4.4 [-5.0; -3.8] | <0.0001 | -7.3 [-8.2; -6.5] | <0.0001 |
| Waist circumference (cm) | 116.0 [113.5; 118.5] | -4.8 [-5.5; -4.1] | <0.0001 | -8.1 [-9.1; -7.0] | <0.0001 |
| Fasting blood glucose (mg/dl) | 126.9 [123.3; 130.5] | -11.1 [-13.0; -9.1] | <0.0001 | -17.6 [-20.0; -15.1] | <0.0001 |
| HbA1c (%) | 6.80 [6.60; 7.00] | -0.35 [-0.46; -0.23] | <0.0001 | -0.55 [-0.65; -0.46] | <0.0001 |
| Systolic blood pressure (mmHg) | 137 [134; 139] | -4.8 [-6.0; -3.7] | <0.0001 | -7.4 [-9.2; -5.7] | <0.0001 |
| Diastolic blood pressure (mmHg) | 83 [81; 85] | -2.4 [-3.3; -1.4] | <0.0001 | -3.6 [-4.8; -2.5] | <0.0001 |
| LDL cholesterol (mg/dl) | 131 [124; 138] | -12.9 [-16.5; -9.3] | <0.0001 | -19.7 [-24.4; -14.9] | <0.0001 |
| HDL cholesterol (mg/dl) | 43.5 [40.9; 46.1] | 3.2 [1.9; 4.5] | <0.0001 | 5.2 [3.7; 6.8] | <0.0001 |
| Triglycerides (mg/dl) | 201 [181; 222] | -30 [-40; -21] | <0.0001 | -41 [-53; -30] | <0.0001 |
| Fasting plasma Insulin (μu/ml) | 25.0 [21.3; 28.7] | -3.5 [-5.9; -1.1] | 0.005 | -5.5 [-8.0; -3.0] | <0.0001 |
| HOMA-IR | 7.3 [6.0; 8.7] | -1.5 [-2.5; -0.6] | 0.002 | -2.5 [-3.5; -1.4] | <0.0001 |
| 10-year cardiovascular risk (%) | 16.7 [12.8; 20.6] | -2.8 [-3.7; -2.0] | <0.0001 | -4.1 [-5.3; -2.9] | <0.0001 |

Supplementary table 4. Estimated marginal means [95% CI] of the parameters of interest at baseline and after 90 days and 180 days of treatment, according to the type of diet (free diet, N=267; balanced diet with -500 Kcal compared to the estimated Total Daily Energy Expenditure, N=238).

| Diet | Baseline | 90 days | 180 days | Estimated mean difference  (balanced diet-free diet) 90 days | P (balanced diet vs. free diet) 90 days | Estimated mean difference  (balanced diet-free diet) 180 days | P (balanced diet vs. free diet) 180 days |
| --- | --- | --- | --- | --- | --- | --- | --- |
| **Weight (kg)** | | | | | | | |
| Free | 90.4 [87.9-93.0] | 86.6 [84.4-88.8] | 84.4 [82.2-86.6] | 1.18 [-4.3;+1.9] | 0.46 | 0.73 [-3.8;+2.3] | 0.64 |
| Balanced | 90.5 [88.2-92.9] | 86.8 [84.6-89.1] | 83.6 [81.5-85.7] |  |  |  |  |
| **Waist circumference (cm)** | | | | | | | |
| Free | 108.1 [105.3-110.9] | 104.5 [101.9-107.1] | 101.9 [99.2-104.5] | 1.25 [-4.6;+2.1] | 0.47 | -0.09[-3.3;+3.4] | 0.96 |
| Balanced | 109.6 [107.2-112.1] | 104.5 [102.1-106.8] | 100.7 [98.3-103.1] |  |  |  |  |
| **Fasting blood glucose (mg/dL)** | | | | | | | |
| Free | 123.5 [120.1-126.8] | 114.5 [111.6-117.5] | 101.5 [98.9-104.0] | -1.74 [-2.4;+5.8] | 0.40 | -4.3 [-8.2;-0.5] | 0.03 |
| Balanced | 121.4 [117.8-125.0] | 109.9 [107.0-112.7] | 100.8 [98.4-103.2] |  |  |  |  |
| **HbA1c (%)** | | | | | | | |
| Free | 6.6 [6.4-6.7] | 6.3 [6.2-6.4] | 6.1 [5.9-6.2] | 0.13 [-0.1;+0.3] | 0.20 | -0.08 [-0.4;+0.3] | 0.67 |
| Balanced | 6.7 [6.6-6.9] | 6.4 [6.2-6.5] | 6.2 [6.0-6.3] |  |  |  |  |
| **Systolic blood pressure (mmHg)** | | | | | | | |
| Free | 135.0 [132.5-137.5] | 130.3 [128.1-132.5] | 128.7 [126.8-130.7] | -0.69 [-3.5;+2.2] | 0.64 | -1.6 [-4.3;+1.0] | 0.23 |
| Balanced | 134.3 [131.9-136.6] | 129.5 [127.6-131.4] | 126.3 [124.6-128.0] |  |  |  |  |
| **Diastolic blood pressure (mmHg)** | | | | | | | |
| Free | 82.9 [81.0-84.9] | 80.9 [79.0-82.8] | 79.1 [77.3-80.9] | -1.4 [-3.5;+0.7] | 0.19 | -1.7 [-3.8;+1.5] | 0.13 |
| Balanced | 81.7 [80.3-83.1] | 78.6 [77.4-79.9] | 77.1 [76.0-78.3] |  |  |  |  |
| **LDL cholesterol (mg/dL)** | | | | | | | |
| Free | 127.4 [120.7-134.0] | 118.9 [113.0-124.9] | 112.5 [106.9-118.0] | 3.7 [-4.4;+11.7] | 0.37 | 2.6 [-5.0;+10.1] | 0.50 |
| Balanced | 134.6 [128.1-141.1] | 121.1 [115.7-126.4] | 113.5 [108.3-118.8] |  |  |  |  |
| **HDL cholesterol (mg/dL)** | | | | | | | |
| Free | 50.7 [45.8-55.5] | 50.9 [47.9-54.0] | 51.8 [48.9-54.7] | -0.94 [-4.8;+2.9] | 0.63 | -0.66 [-4.1;+2.8] | 0.70 |
| Balanced | 48.8 [46.0-51.6] | 51.4 [48.9-53.9] | 53.1 [50.9-55.4] |  |  |  |  |
| **Triglycerides (mg/dL)** | | | | | | | |
| Free | 181.6 [164.4-198.7] | 160.2 [146.7-173.7] | 152.6 [140.2-164.9] | 9.0 [-10.2;+28.3] | 0.36 | -1.8 [-17.0;+13.4] | 0.81 |
| Balanced | 187.9 [174.2-201.6] | 163.4 [143.4-183.4] | 141.1 [133.5-148.7] |  |  |  |  |
